# The dynamic DNA methylation landscape of the *mutL homolog 1* shore is altered by *MLH1*-93G>A polymorphism in normal tissues and colorectal cancer

**DOI:** 10.1186/s13148-017-0326-6

**Published:** 2017-03-09

**Authors:** Andrea J. Savio, Miralem Mrkonjic, Mathieu Lemire, Steven Gallinger, Julia A. Knight, Bharat Bapat

**Affiliations:** 1 Lunenfeld-Tanenbaum Research Institute, Sinai Health System, 60 Murray St., Toronto, Ontario M5T 3L9 Canada; 2 Department of Laboratory Medicine and Pathobiology, Faculty of Medicine, University of Toronto, 27 King’s College Circle, Toronto, Ontario M5S 1A1 Canada; 3 Ontario Institute for Cancer Research, 661 University Avenue, Toronto, Ontario M5G 0A3 Canada; 4 Ontario Familial Colorectal Cancer Registry, Cancer Care Ontario, 60 Murray St., Toronto, Ontario M5T 3L9 Canada; 5 Department of Surgery, University Health Network, 200 Elizabeth St., Toronto, ON M5G 2C4 Canada; 6 Dalla Lana School of Public Health, University of Toronto, 155 College St., Toronto, ON M5T 3M7 Canada; 7 Department of Pathology, University Health Network, 200 Elizabeth St., Toronto, ON M5G 2C4 Canada

**Keywords:** Colorectal cancer, *mutL homolog 1*, Single nucleotide polymorphism, DNA methylation, Epigenetics, MethyLight, Mismatch repair, Microsatellite instability, CpG island, CpG shore

## Abstract

**Background:**

Colorectal cancers (CRCs) undergo distinct genetic and epigenetic alterations. Expression of *mutL homolog 1* (*MLH1*), a mismatch repair gene that corrects DNA replication errors, is lost in up to 15% of sporadic tumours due to mutation or, more commonly, due to DNA methylation of its promoter CpG island. A single nucleotide polymorphism (SNP) in the CpG island of *MLH1* (*MLH1*-93G>A or rs1800734) is associated with CpG island hypermethylation and decreased *MLH1* expression in CRC tumours. Further, in peripheral blood mononuclear cell (PBMC) DNA of both CRC cases and non-cancer controls, the variant allele of rs1800734 is associated with hypomethylation at the *MLH1* shore, a region upstream of its CpG island that is less dense in CpG sites*.*

**Results:**

To determine whether this genotype-epigenotype association is present in other tissue types, including colorectal tumours, we assessed DNA methylation in matched normal colorectal tissue, tumour, and PBMC DNA from 349 population-based CRC cases recruited from the Ontario Familial Colorectal Cancer Registry. Using the semi-quantitative real-time PCR-based MethyLight assay, *MLH1* shore methylation was significantly higher in tumour tissue than normal colon or PBMCs (*P* < 0.01). When shore methylation levels were stratified by SNP genotype, normal colorectal DNA and PBMC DNA were significantly hypomethylated in association with variant SNP genotype (*P* < 0.05). However, this association was lost in tumour DNA. Among distinct stages of CRC, metastatic stage IV CRC tumours incurred significant hypomethylation compared to stage I–III cases, irrespective of genotype status. Shore methylation of *MLH1* was not associated with MSI status or promoter CpG island hypermethylation, regardless of genotype. To confirm these results, bisulfite sequencing was performed in matched tumour and normal colorectal specimens from six CRC cases, including two cases per genotype (wildtype, heterozygous, and homozygous variant). Bisulfite sequencing results corroborated the methylation patterns found by MethyLight, with significant hypomethylation in normal colorectal tissue of variant SNP allele carriers.

**Conclusions:**

These results indicate that the normal tissue types tested (colorectum and PBMC) experience dynamic genotype-associated epigenetic alterations at the *MLH1* shore, whereas tumour DNA incurs aberrant hypermethylation compared to normal DNA.

## Background

Colorectal cancer (CRC) develops as a result of the accumulation of genetic and epigenetic alterations. Aberrant hypermethylation of CpG islands along with genome-wide hypomethylation is a common signature in CRC [1, 2]. While a number of genes have been shown to incur methylation in CRC, one of the best studied of these is the DNA mismatch repair (MMR) gene *mutL homolog 1* (*MLH1*) [3–6]. Loss of *MLH1* or other MMR genes leads to the accumulation of mutations, particularly at repetitive microsatellite regions leading to microsatellite instability (MSI) [6–8]. Approximately 15% of CRCs exhibit the MSI-high (MSI-H) phenotype, and the majority of these cases have deficient MMR function due to hypermethylation incurred at the *MLH1* promoter CpG island [5, 9].

Germline mutations of *MLH1* or other MMR genes, including *mutS homolog 2* (*MSH2*), *mutS homolog 6* (*MSH6*), and *PMS1 homolog 2, mismatch repair system component* (*PMS2*), lead to Lynch Syndrome accounting for approximately 2–5% of CRCs [10, 11]. Mutations in *APC* cause familial adenomatous polyposis, occurring in <1% of CRCs [12, 13]. While these and several other rarer germline gene mutations are known contributors to ~10% of CRCs, twin and family studies have estimated the heritability of CRC to be up to 35% [14]. Single nucleotide polymorphisms (SNPs) have been estimated to account for at least 7.42% of this heritability [15].

A number of genome-wide association studies (GWAS) have established susceptibility loci for CRC, including at 8q24, 11q23, and others [16–20]. It has previously been demonstrated that a SNP in the promoter CpG island of *MLH1* (*MLH1*-93G>A, rs1800734) is associated with *MLH1* CpG island hypermethylation, loss of protein expression, MSI, and overall increased risk of MSI-H CRC [21, 22]. A subsequent study implicated the variant A allele of the SNP as contributing to increased risk of CRC overall, though another study refuted this [23, 24]. While the overall status of this SNP as a risk factor for CRC needs further clarification, what is clear is that it plays a role in MSI-H CRC and *MLH1* CpG island methylation status. Interestingly, further study of SNP rs1800734 in peripheral blood mononuclear cell (PBMC) DNA using Illumina 450K methylation arrays indicated a different phenomenon occurring upstream of this SNP and the CpG island in which it is located. At the *MLH1* shore in PBMCs of both CRC cases and controls, we observed significant hypomethylation in association with variant SNP genotype [25].

Although the majority of DNA methylation research has focused on CpG islands, whole-genome methylation studies have shown that methylation changes at other non-coding regulatory regions such as CpG shores and enhancers may also be implicated in tumourigenesis [26, 27]. CpG shores are regions flanking some CpG islands that are less dense in CpG dinucleotides than the corresponding islands are. Differential shore methylation has been shown to discriminate between normal and tumour DNA in colorectal, prostate, and breast cancer, among other diseases [26, 28, 29].

While the mechanisms that direct DNA methylation patterns are not yet completely understood, it is guided at least in part by DNA sequence [30–33]. We have previously demonstrated DNA variant-associated CpG shore hypomethylation in PBMCs while CpG island hypermethylation was shown in CRC tumours, both of which occur in association with the same single nucleotide change [22, 25]. In this study, DNA methylation of the *MLH1* shore was investigated in a large cohort of 349 population-based CRC cases to determine its association with rs1800734 SNP genotype in normal colorectal tissue, colorectal tumours, and PBMCs of the same patients. These results indicate that static genetic variants can dynamically modulate epigenetic regulation at the *MLH1* gene region and may play a role in colorectal tumourigenesis.

## Methods

### Study subjects

Participants in this study were recruited through the Ontario Familial Colorectal Cancer Registry (OFCCR), which is part of the Colon Cancer Family Registry, a consortium supported by the US National Cancer Institute. Recruitment of primary CRC cases and controls was population-based and has been described previously [34]. Briefly, residents from Ontario, Canada, diagnosed with primary CRC between June 1, 1997, and June 30, 2000, between the ages of 20 and 74 were eligible for recruitment during phase I. For phase II, individuals with incident CRC under the age of 50 diagnosed in Ontario between January 2003 and December 2006 were recruited. Additional clinic-based recruitment was performed to recruit individuals diagnosed with CRC above the age of 49 with fresh frozen tumour specimens available at the biospecimen repository. Familial adenomatous polyposis cases were excluded from both phase I and II. Cases with non-white, mixed ethnic, or unknown background were excluded from the current study due to the high proportion of self-reported Caucasians. Participants provided blood, tumour, and non-neoplastic colorectal mucosa samples, henceforth referred to as normal colorectal mucosa. These blood and tissue samples were obtained with informed written consent following protocols approved by the research ethics board of Mount Sinai Hospital and the University of Toronto.

### Single nucleotide polymorphism genotyping

SNP selection and genotyping has been previously described [22]. Briefly, PBMCs were isolated from blood samples of cases and controls by Ficoll-Paque gradient centrifugation following manufacturer’s protocol (Amersham Biosciences, Baie d’Urfé, Quebec, Canada). DNA was extracted from PBMCs by phenol-chloroform or Qiagen DNA extraction kit (Qiagen Inc., Hilden, Germany). The SNP rs1800734 was genotyped using a fluorogenic 5ʹ nuclease polymerase chain reaction (PCR) assay. It was also genotyped using Affymetrix GeneChip Human Mapping 100K and 500K platforms through the Assessment of Risk of Colorectal Tumours in Canada project [35, 36]. Genotypes of the five OFCCR phase II samples used for bisulfite sequencing were confirmed by Sanger sequencing at The Centre for Applied Genomics (TCAG), The Hospital for Sick Children, Toronto, Canada. DNA from samples was amplified by PCR for the region in the *MLH1* promoter encompassing rs1800734 using primer sequences: (forward) 5ʹ-CGCCACATACCGCTCGTAGTA-3ʹ and (reverse) 5ʹ-TCCGTACCAGTTCTCAATCATCTC-3ʹ. Sequencing was performed at TCAG using an internal primer (forward) 5ʹ-GTCATCCACATTCTGCGGGA-3ʹ.

### Microsatellite instability analysis

PCR was performed on tumour and matched normal colorectal tissue DNA to compare MSI patterns as described previously [35]. Briefly, paraffin-embedded colorectal tumour tissue and normal colorectal tissue from the same patients were microdissected for areas with more than 70% cellularity in tumour and normal cell populations. The MSI status was determined by using the National Cancer Institute guidelines, assessing four or more markers of ACTC, BAT-25, BAT-26, BAT-40, BAT-34C4, D10S197, D18S55, D17S250, D5S346, and MYC-L. MSI status was defined as MSI-high (MSI-H) if ≥30% of markers were unstable; MSI low (MSI-L) if 1–29% of markers were unstable; and microsatellite stable (MSS) if 0% of markers were unstable [36].

### MethyLight

MethyLight was used to determine the DNA methylation status of the *MLH1* shore in PBMCs, normal colorectal tissue, and colorectal tumours of CRC cases. Fifty nanograms of DNA was subject to bisulfite modification with the EZ DNA Methylation Gold Kit according to manufacturer’s protocol (Zymo Research Corp., Orange, CA). Primers and probe were used to amplify a region of the *MLH1* shore, with *ALU-C4* primers and probe used as control. Probes contained a 5ʹ fluorescent reporter dye and a 3ʹ quencher dye. Sequences for the *MLH1* shore are as follows: (forward) 5ʹ-ATAGTTTTGATTAAGATTAGAGGCG-3ʹ, (reverse) 5ʹ-CGATGTTTGAATAATTGGTTTAGG-3ʹ, and (probe) 5ʹ-AGGCGATTTGAATTTTAGATTTTATTAACGGAA-3ʹ. Sequences for *ALU-C4* are as follows: (forward) 5ʹ-GGTTAGGTATAGTGGTTTATATTTGTAATTTTAGTA-3ʹ, (reverse) 5ʹ-ATTAACTAAACTAATCTTAAACTCCTAACCTCA-3ʹ and (probe) 5ʹ-CCTACCTTAACCTCCC-3ʹ. Samples were analysed in duplicate in 96-well plates on an ABI 7500 RT-PCR thermocycler. Percent methylated reference (PMR) score was calculated using the following calculation: [Gene of Interest/*ALU-C4*]_sample_/[Gene of Interest/*ALU-C4*]_CpGenome_ × 100%, where CpGenome represents commercially available fully methylated CpGenome Universal Methylated DNA (Millipore, Billerica, MA). The cases selected for MethyLight profiling in this study were those from phase I OFCCR genotyped for SNP rs1800734 with available peripheral blood mononuclear cell, non-neoplastic colorectal mucosa, and tumour DNA. *MLH1* CpG island methylation was determined previously using MethyLight for these cases in the same manner [22].

### Bisulfite sequencing

Bisulfite sequencing was performed to analyse DNA methylation in tumour and matched normal colorectal mucosa from six CRC cases. Genomic DNA from formalin-fixed paraffin-embedded (one case from phase I OFCCR) or fresh frozen (five cases from phase II OFCCR) tissue was used. DNA was treated with EZ DNA Methylation Gold Kit for bisulfite conversion, as described previously. Primers located within the *MLH1* shore were designed as follows: (forward) 5ʹ-TTTGTTTGAGAAGTGGATTGTTGTTG-3ʹ and (reverse) 5ʹ-TTTCTTCACTTAAAACTATTAAACTCC-3ʹ. DNA was amplified by PCR for each tumour and normal colorectal sample. PCR product was purified using ChargeSwitch PCR Clean-Up Kit (Invitrogen). PCR products were cloned using the pGEM-T Easy Vector System (Promega, Madison, WI) and MAX Efficiency DH5α Competent Cells (Life Technologies, Carlsbad, CA) according to manufacturer’s protocol. QIAprep Spin Miniprep Kit was used according to protocol to extract plasmid DNA (Qiagen, Hilden, Germany). Each successful clone was sequenced by Sanger sequencing at TCAG and at least 15 clones were sequenced for each sample.

### Statistical analysis

Sex, stage, and MSI status were compared between the cases utilized for this study and the entire OFCCR cohort by Pearson’s chi-square tests. Age was compared between the two groups by Independent samples *T* tests. MethyLight PMR values were utilized to build a multiple linear regression model to assess the relationship between methylation level at the *MLH1* shore and tissue type (PBMC, normal colorectal mucosa, tumour), rs1800734 genotype, age, sex, stage (TNM stage I–III compared to IV), MSI status, and *MLH1* CpG island methylation status. MethyLight PMR values among tissue type were compared using a linear mixed model, controlling for random and fixed effects to account for different DNA sources from the same individual. PMR values between genotypes were compared using ANOVA and independent samples *T* tests. Independent samples *T* tests were used to compare methylation with clinicopathological variables of cases. A 6 × 4 contingency table and Pearson’s chi-square tests were used to compare the sum of methylated CpGs between genotypes for each sample (total of 6 CpGs per clone with 15–27 clones per sample). All tests were performed using IBM SPSS Statistics 21 with two-sided *P* < 0.05 defined as statistically significant.

## Results

### Assessment of clinicopathological variables and DNA methylation

Genotype and clinicopathological variables for the 349 CRC cases used in this study are shown in Table 1. The cases utilized for this project constitute only a subset of the total cases recruited for the OFCCR. The 349 cases selected had been previously profiled on the Illumina Infinium HumanMethylation450 arrays, had known rs1800734 genotype status, and had available DNA from PBMC, normal colorectal mucosa, and tumour tissue. These cases did not differ significantly from the entire cohort of cases by age, sex, stage, or MSI status.

**Table 1:** Distribution of clinicopathological features in primary colorectal carcinomas, including distribution among genotypes of rs1800734

Feature	All Genotypes	GG	GA	AA
	*N* (%)	*N* (%)	*N* (%)	*N* (%)
Cases of primary colorectal carcinoma	349	211 (60.5)	119 (34.1)	19 (5.4)
Mean age (±SD)	61.9 (8.8)	62.0 (9.1)	62.0 (8.4)	60.8 (8.1)
Sex				
Female	163 (46.7)	95 (45.0)	58 (48.7)	11 (57.9)
Male	186 (53.3)	116 (55.0)	61 (51.3)	8 (42.1)
TNM stage				
1	22 (6.3)	15 (7.1)	6 (5.0)	1 (5.3)
2	81 (23.2)	52 (24.6)	27 (22.7)	2 (10.5)
3	208 (59.6)	125 (59.2)	70 (58.8)	13 (68.4)
4	21 (6.0)	12 (5.7)	8 (6.7)	1 (5.3)
Unavailable	17 (4.9)	7 (3.3)	8 (6.7)	2 (10.5)
MSI status				
Stable/low	287 (82.2)	181 (85.8)	95 (79.8)	11 (57.9)
High	62 (17.8)	30 (14.2)	24 (20.2)	8 (42.1)
*MLH1* CpG island				
Unmethylated	300 (86.0)	187 (88.6)	98 (82.3)	15 (78.9)
Methylated	34 (9.7)	17 (8.1)	14 (11.8)	3 (15.8)
Unavailable	15 (4.3)	7 (3.3)	7 (5.9)	1 (5.3)
MMR germline mutation				
No	341 (97.7)	209 (99.1)	114 (95.8)	18 (94.7)
Yes	8 (2.3)	2 (0.9)	5 (4.2)	1 (5.3)

Percentages may not add up to 100.0 due to rounding

*SD* standard deviation

A multiple linear regression model was built to examine the relationships between methylation levels at the *MLH1* shore and rs1800734 genotype, age, sex, tumour stage, MSI status, and *MLH1* CpG island methylation status (Table 2). Genotype was significantly associated with methylation (*r =* −0.07, *P* = 0.045). Examination of the relationships between methylation and clinicopathological variables within each tissue type was also performed. *MLH1* shore methylation within PBMC DNA was significantly associated with rs1800734 genotype (*r =* −0.14, *P* = 0.02). Within normal colorectal tissue DNA, shore methylation was associated with SNP genotype (*r =* −0.15, *P* = 0.01), age (*r =* 0.19, *P* = 0.001), and tumour stage (*r = *0.13, *P* = 0.04). Tumour DNA methylation was associated with tumour stage (*r = *-0.14, *P* = 0.02).

**Table 2:** Multiple linear regression model for all DNA sources as well as PBMC, normal colorectal mucosa, and CRC tumour DNA

	All DNA sources		PBMC		Normal colon		Tumour	
Variable	*r*	*P*	*r*	*P*	*r*	*P*	*r*	*P*
Tissue	0.06	0.07						
rs1800734	−0.07	*0.045*	−0.14	*0.02*	−0.15	*0.01*	0.03	0.58
Age	0.07	0.05	−0.05	0.28	0.19	*0.001*	0.04	0.36
Sex	0.0002	0.78	0.06	0.47	0.003	0.77	−0.04	0.54
Stage	−0.01	0.79	0.11	0.07	0.13	*0.04*	−0.14	*0.02*
MSI status	0.03	0.93	0.05	0.48	0.04	0.91	0.01	0.70
*MLH1* CpG island methylation	−0.03	0.49	−0.02	0.60	−0.02	0.57	−0.04	0.39

Significant results are italicized

The Pearson correlation (*r*) and *P* value for each variable are described

### The *MLH1* shore is hypermethylated in tumour DNA

**Fig. 1:**
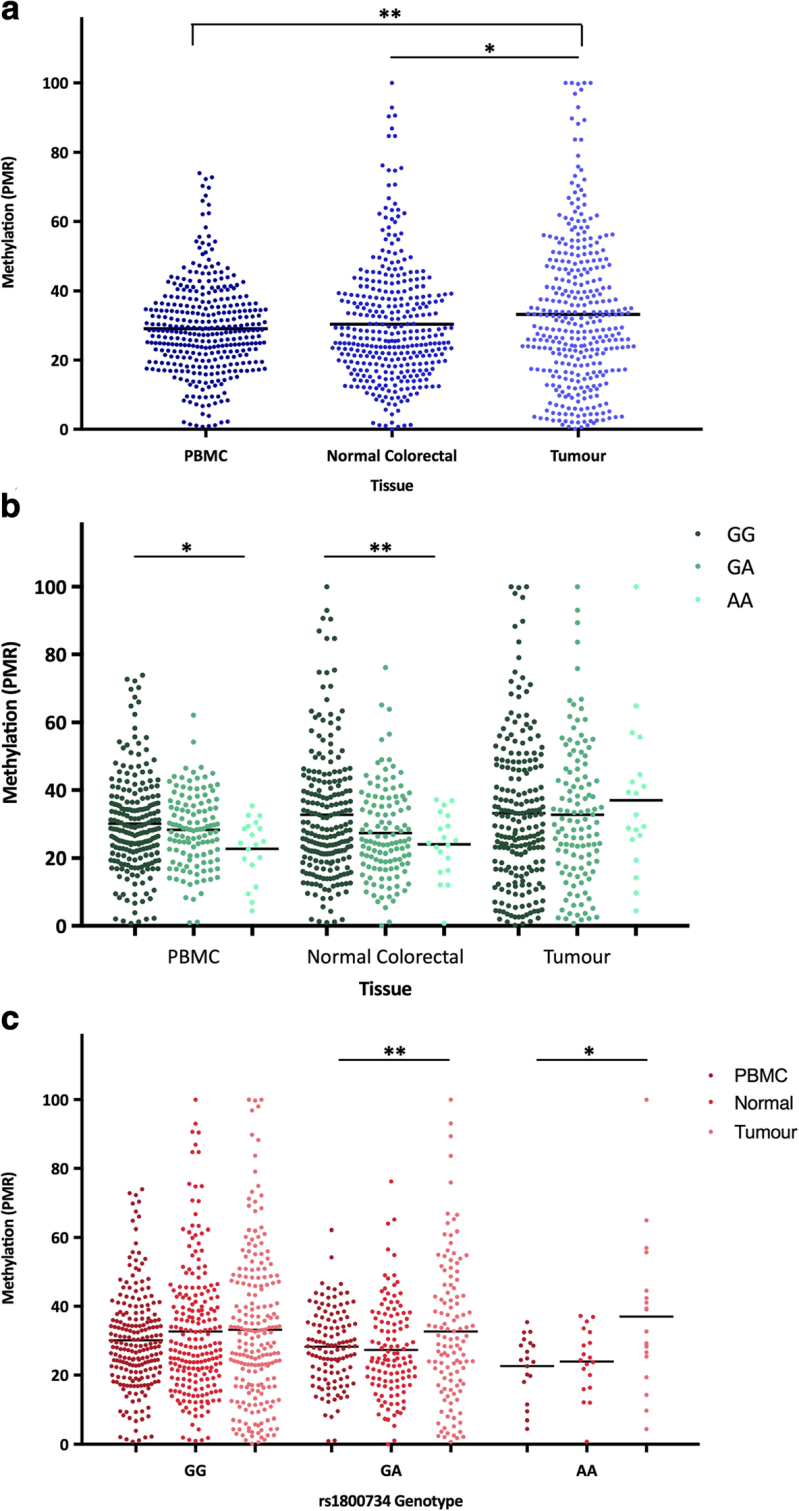
Mean percent methylated reference (PMR) of the *MLH1* shore. MethyLight was utilized to determine PMR in PBMC, normal colorectal tissue, and colorectal tumour DNA of 349 population-based CRC cases. All cases were genotyped for SNP rs1800734. There were 211 wildtype (GG), 119 heterozygous (GA), and 19 homozygous variant (AA) carriers. **a** Mean *MLH1* shore methylation in each DNA source. **b** Mean *MLH1* shore methylation in each DNA source stratified by genotype of rs1800734. **c** Mean *MLH1* shore methylation for each genotype of rs1800734 stratified by DNA source. *P < 0.05 and **P < 0.01

MethyLight was performed to measure methylation at the *MLH1* shore in DNA extracted from PBMCs, normal colorectal tissue, and colorectal tumours of 349 CRC patients. Mean methylation was compared using a linear mixed model across the three tissue types that were analysed (Fig. 1a). Mean PMR [standard deviation (SD)] in PBMCs was 29.1% (4.5), compared to 30.5% (5.8) in normal colorectal mucosa, and 33.3% (7.2) in tumour. *MLH1* shore methylation was significantly higher in tumour than normal colorectal tissue (*P* = 0.04) and PBMCs (*P* = 0.001). Mean methylation did not differ significantly between normal colorectal tissue and PBMCs (*P* = 0.22).

### The *MLH1* shore is hypomethylated in variant SNP carriers in normal DNA

Mean methylation at the *MLH1* shore was compared between each genotype of rs1800734 in PBMCs, normal colorectal tissue, and colorectal tumours by ANOVA (Fig. 1b). In PBMCs, mean methylation (SD) of GG, GA, and AA cases was 30.1% (4.8), 28.3% (3.6), and 22.6% (3.0), respectively. This SNP-associated hypomethylation was significant (*P* = 0.04). Comparing individual genotypes, the methylation between GG and AA genotypes as well as between GA and AA genotypes in PBMC DNA was also significantly different (*P =* 0.003 and 0.02, respectively). These findings in PBMC DNA utilizing RT-PCR-based MethyLight technique confirmed our previously published Illumina array-based results indicating SNP-associated hypomethylation of the *MLH1* shore region [25]. Comparing methylation among genotypes in normal colorectal tissue, mean methylation (SD) of GG, GA, and AA cases was 32.8% (6.5), 27.3% (4.5), and 24.0% (3.2), respectively, and these results were also significant (*P* = 0.005). Comparing individual genotypes, the shore methylation between GG and GA genotypes as well as between GG and AA genotypes was also significantly different (*P =* 0.003 and 0.002, respectively). In tumour samples stratified by genotype, mean methylation (SD) of individuals with the wildtype GG genotype was 33.3% (7.4), while in GA individuals it was 32.8% (7.0), and 37.0% (7.3) in AA individuals. Methylation did not differ significantly in tumour DNA of CRC cases regardless of SNP rs1800734 genotype, either by all three genotypes or individual genotype comparisons (e.g., GG vs. GA) (*P* > 0.05).

### Tumour hypermethylation at the *MLH1* shore is driven by variant SNP allele

Mean methylation was compared within each genotype of rs1800734, shown in Fig. 1c, to examine the methylation patterns across normal and tumour DNA at the *MLH1* shore. Among individuals with the wildtype GG genotype of rs1800734, mean methylation (SD) was 30.1% (4.8) in PBMCs, 32.8% (6.5) in normal colorectal tissue, and 33.3% (7.4) in tumour tissue, which did not differ significantly (*P* = 0.15). In heterozygous individuals carrying the GA genotype, mean methylation (SD) was 28.3% (3.6) in PBMCs, 27.3% (4.5) in normal colorectal tissue, and 32.8% (7.0) in tumour, which differs significantly between tissues (*P* = 0.008). Lastly, in homozygous variant individuals carrying the AA genotype, mean methylation (SD) was 22.6% (3.0) in PBMCs, 24.0% (3.2) in normal colorectal tissue, and 37.1% (7.3) in tumour tissue, which also varied significantly between tissues (*P* = 0.01). Overall, significant hypermethylation in tumours compared to normal DNA is incurred only in individuals carrying one or two variant alleles of rs1800734, either GA or AA.

### The *MLH1* shore is hypomethylated in stage IV CRC, not associated with CpG island methylation or MSI

**Fig. 2:**
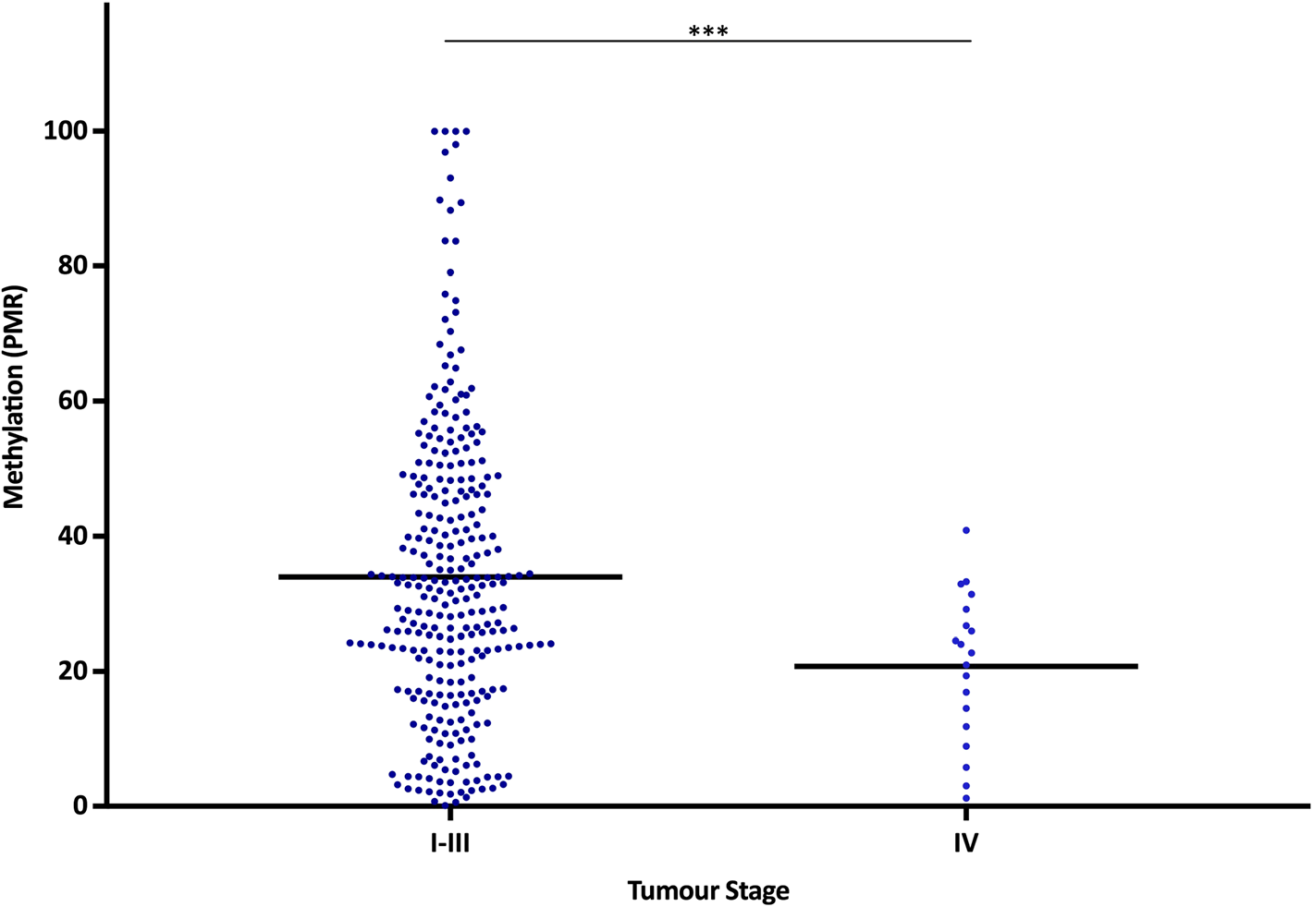
Bisulfite sequencing of the *MLH1* shore in normal colorectal tissue and matched colorectal tumours. **a** The *MLH1* upstream region is indicated, including rs1800734 coordinates. The position of each CpG interrogated by bisulfite sequencing relative to the *MLH1* translation start site is designated by a *circle*. Each CpG is numbered 1 to 6 in a 5ʹ to 3ʹ direction. The *grey circles* represent CpG sites also interrogated within the MethyLight amplicon. **b** Methylation patterns in six normal colorectal tissue samples and matched tumours at the *MLH1* shore, with rs1800734 genotype indicated. *Empty circles* represent unmethylated CpGs and *filled circles* represent methylated CpGs. **c** Graphical representation of bisulfite sequencing results. For each sample at each CpG site, the percent of methylated CpGs was calculated. The mean percent of methylated CpGs was then calculated for each genotype and tissue source grouping: GG normal, GA normal, AA normal, GG tumour, GA tumour, and AA tumour. Pearson’s chi-square test was used to compare the total number of methylated CpGs at each CpG site. *Error bars* represent standard deviation. **P < 0.01; ***P < 0.001

*MLH1* shore methylation in tumours was tested for associations with various clinicopathological variables of the 349 CRC cases that were assessed by MethyLight. Tumour stage and *MLH1* shore methylation in tumour DNA was compared. The mean methylation (SD) of stage I–III tumours was 34.1% (7.3) versus 20.8% (3.6) in stage IV cases, which was highly significantly different (*P* = 6.2 × 10^−5^) (Fig. 2). Hypomethylation in stage IV cases was apparent for all cases, regardless of SNP genotype. In GG cases mean methylation was 33.6% (7.4) for stage I–III and 22.4% (4.3) for stage IV (*P* = 0.02). In GA cases mean methylation was 33.7% (7.1) for stage I–III and 18.4% (2.4) for stage IV (*P* = 0.001). There was only one stage IV case with the AA genotype thus no *P* value was calculated.

Mean methylation (SD) in tumours of cases with MSI-H phenotype was 32.9% (5.7), which did not differ from the 33.2% (7.5) observed in MSS/MSI-L cases (*P* = 0.90). When stratified by rs1800734 SNP genotype, methylation still did not differ significantly between MSI-H and MSS/MSI-L cases (all *P* > 0.05). We next tested whether or not methylation of the *MLH1* CpG island in tumour DNA was associated with *MLH1* shore methylation in tumour DNA of the same individuals. Cases were considered methylated at the *MLH1* CpG island if PMR was greater or equal to 10%, as has been previously established [22, 37]. Mean *MLH1* shore methylation was 33.4% (7.4) in tumour DNA of cases that were unmethylated at the island while methylation was 30.4% (5.9) at the shore of cases that were methylated at the CpG island, which did not differ significantly (*P* = 0.41). Since it has previously been demonstrated that *MLH1* CpG island hypermethylation is associated with rs1800734 variant genotype, we assessed whether shore methylation was associated with CpG island methylation stratified by genotype; however, there were no significant associations (all *P* > 0.05). Thus, regardless of genotype, *MLH1* shore methylation is not associated with CpG island methylation or MSI status.

*MLH1* shore methylation level in PBMCs or normal colorectal tissue was not significantly associated with MSI status, tumour *MLH1* CpG island hypermethylation, or tumour stage for all cases or when stratified by SNP genotype of rs1800734 (all *P* > 0.05).

### Bisulfite sequencing confirms SNP-associated hypomethylation of *MLH1* shore in normal colorectal DNA

**Fig. 3:**
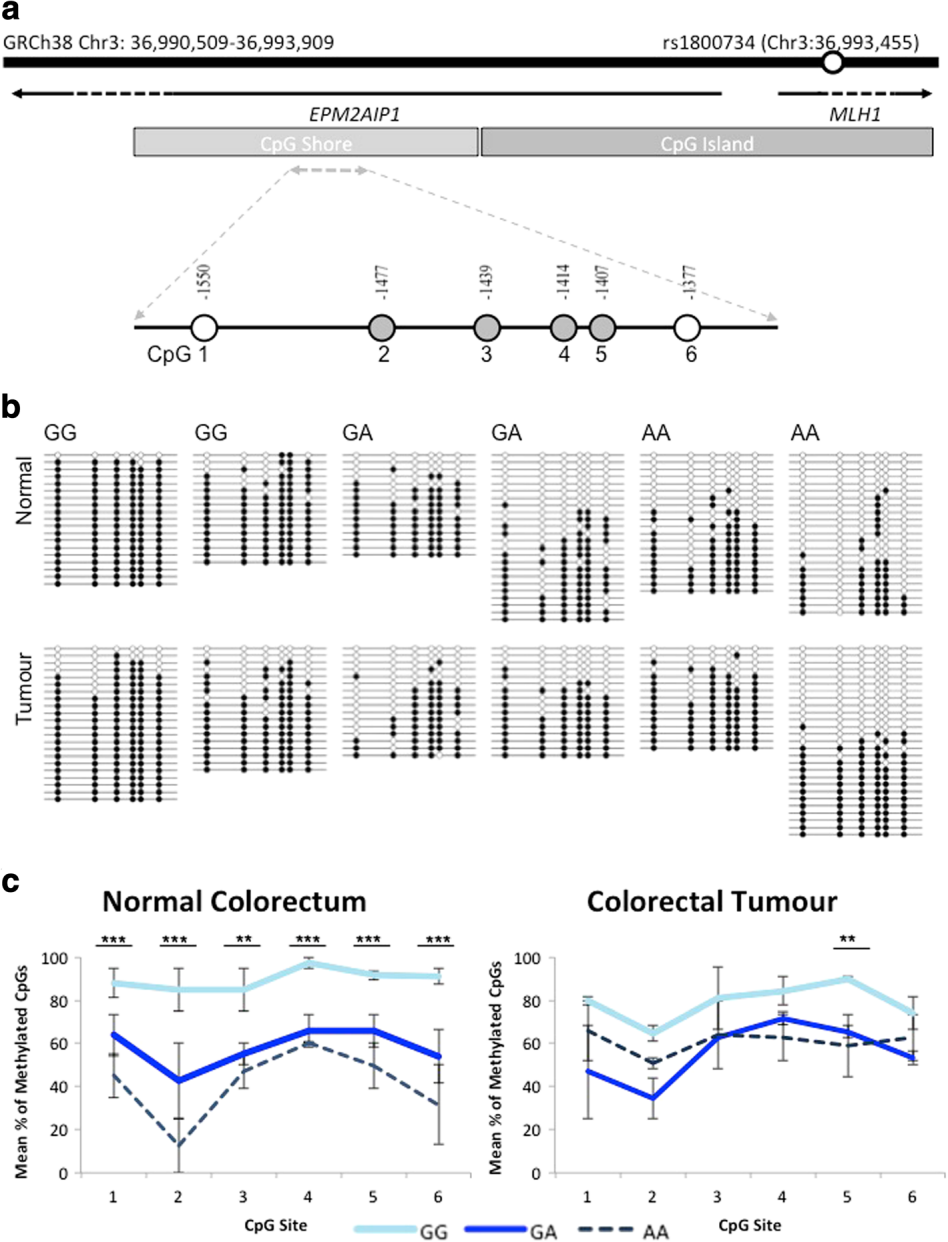
Mean percent methylated reference (PMR) of the *MLH1* shore among different stages in tumour DNA. MethyLight was utilized to determine PMR in colorectal tumour DNA of 349 population-based CRC cases. Mean *MLH1* shore methylation among stage I–III and stage IV cases is indicated. ***P < 1.0 × 10^−4^

Bisulfite sequencing was performed on a 232-base-pair region of the *MLH1* shore containing six CpGs, which we refer to as CpG 1 to 6 in a 5ʹ to 3ʹ direction (Fig. 3a). CpGs 2–5 were included in the MethyLight primer and probe sequences. Normal colorectal DNA and tumour DNA was analysed from six CRC cases, comprising of two samples with each genotype (GG, GA, and AA). For each sample, 15 to 27 clones were sequenced, and the methylation patterns are shown in Fig. 3b. Visual inspection of the methylation patterns shows hypomethylation in normal colorectal DNA in GA and AA cases compared to GG.

The total number of methylated CpGs at each site was compared between genotypes for normal and tumour samples, shown in Fig. 3c. All genotypes were compared against each other at CpGs 1 to 6 in normal colorectal tissue. DNA methylation as assessed by bisulfite sequencing was statistically significantly different among the three genotypes. *P* values for each of the six CpG sites were CpG 1 *P* = 2.7 × 10^−4^; CpG 2 *P* = 3.2 × 10^−10^; CpG 3 *P* = 0.001; CpG 4 *P* = 5.0 × 10^−4^; CpG 5 *P* = 3.5 × 10^−4^; and CpG 6 *P* = 3.6 × 10^−7^. These results follow the same significant pattern that was observed using the MethyLight technique (Fig. 1b); normal colorectal tissue incurs hypomethylation at the *MLH1* shore in individuals with variant rs1800734 genotype.

The number of methylated CpGs was compared between genotypes in tumour samples. Comparing GG vs. GA vs. AA genotypes, CpG 5 was significantly differentially methylated (*P* = 0.002). Though CpG 5 showed differential methylation, all other CpG site comparisons between genotypes were not significantly statistically different. This corresponds with the MethyLight findings, in which tumour DNA methylation of the *MLH1* shore did not differ among genotypes of rs1800734. Methylation was also compared between normal and tumour DNA, and methylation at none of the six sites was significantly different (all *P* > 0.05).

## Discussion

Aberrant methylation changes are a hallmark of all cancers, including CRC. Though the general pattern observed in tumour DNA includes CpG island hypermethylation with genome-wide hypomethylation, the findings of the present study demonstrate that methylation may be altered in a tissue-, locus-, and genotype-specific manner. The critical mismatch repair gene *MLH1* incurs CpG island hypermethylation in a subset of CRC cases. We have investigated its upstream shore and determined that there is dynamic interplay between genotype and epigenotype in normal and tumour DNA of CRC patients. In normal colorectal tissue, DNA methylation is present at the CpG shore but is hypomethylated in individuals carrying one or two variant alleles of the rs1800734 SNP. This validates our previous array-based results in PBMC DNA of the same CRC cases as well as controls [25]. We also found that this SNP-associated hypomethylation pattern is lost in tumour DNA due to increases in CpG shore methylation in tumour compared to normal colorectal DNA of SNP variant carriers. These findings establish that the static genetic sequence can modulate epigenetic marks in normal tissues. These results also provide further evidence of shore methylation changes in matched tumour versus normal DNA.

**Fig. 4:**
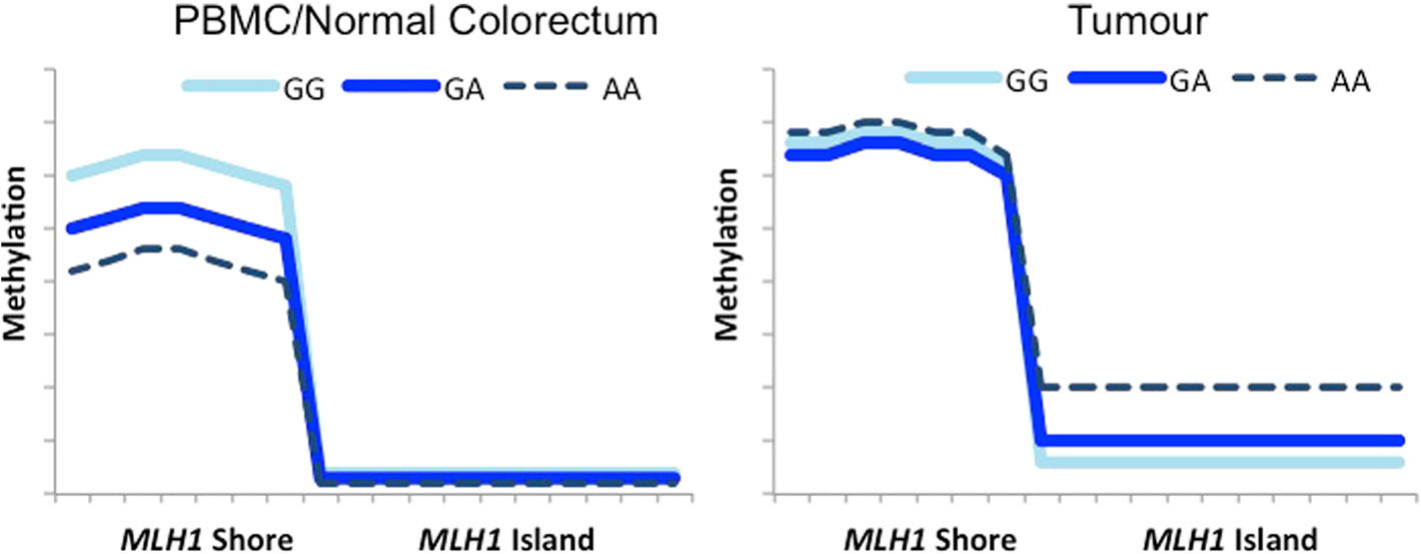
Schematic model of DNA methylation at the *MLH1* CpG island and shore. In PBMCs and normal colorectal tissue (*left panel*), the *MLH1* shore incurs hypomethylation in association with variant SNP genotype of rs1800734. No methylation is present at the CpG island in these DNA sources. In colorectal tumour (*right panel*), DNA methylation at the CpG shore loses its association with rs1800734 genotype whereas the CpG island incurs hypermethylation in association with variant SNP genotype of rs1800734

This study delved into DNA methylation patterns at the *MLH1* shore, which builds upon previous studies of *MLH1* and its promoter SNP. Variant rs1800734 was first shown to be associated with MSI-H CRCs, then with *MLH1* CpG island hypermethylation [21, 22]. Subsequent studies have also shown an association between this SNP and endometrial and lung cancer risk, as well as worse outcome in oral squamous cell carcinoma [38–42]. Our results have revealed the methylation patterns among rs1800734 genotypes at the *MLH1* shore in PBMCs, normal colorectal tissue, and colorectal tumour tissue. We have also previously interrogated *MLH1* CpG island methylation in PBMC, normal colorectal, and tumour DNA [22, 25]. Figure 4 integrates methylation data from the island and shore of *MLH1* to demonstrate the shifting epigenetic patterns at the *MLH1* region*.* SNP genotype is associated with the opposite direction of methylation at the CpG shore and island in normal and tumour DNA.

CpG shores were first described as regions up to 2 kilobases away from CpG islands that are less dense in CpGs [26]. This original publication and subsequent studies have demonstrated that shore methylation differs between different tissue types [26, 43, 44]. However, in the two ‘normal’ DNA sources assessed, non-neoplastic colorectal mucosa and PBMCs, there were no significant differences in *MLH1* shore methylation. Having only examined two sources of non-cancer tissues, it cannot be said for certain what methylation patterns would be seen in other normal tissues from these patients at this specific region. It has also been shown that methylation significantly differs between normal and matched tumour DNA at CpG shore regions in multiple cancer types [26, 28, 29, 45]. The results in this study have indicated tumour hypermethylation at the *MLH1* shore, agreeing with other reports of shore hypermethylation in cancer. Another key feature of shores is that they have been shown to have a stronger negative correlation between methylation and gene expression than CpG islands [26]. However, it was also found that certain subsets of genes with unmethylated islands and methylated shores, termed ‘ravines’, in fact had high transcriptional activity and a more transcriptionally permissive state including higher DNase sensitivity and RNA polymerase II binding [46]. In normal DNA and CRC tumours without *MLH1* CpG island hypermethylation, a similar pattern is seen, with methylation at the shore and no methylation at the CpG island. In fact, the *MLH1* promoter region has been well characterized, and the link between decreased expression and hypermethylation at specific regions in its CpG island have already been established [47, 48]. Thus, methylation at the *MLH1* shore likely does not play a large role, if any, in *MLH1* expression though perhaps it functions in other ways to create a transcriptionally permissive state as in the aforementioned ‘ravines’ [46]. The exact mechanism or functional role for shores remains to be elucidated.

Much research has been focused toward discerning disease-associated SNPs. The majority of SNPs mapped in GWAS are located in non-coding regions of the genome, thus establishing the function of SNPs has been difficult [49–51]. Rather than altering protein function, it has been postulated that variant SNPs cause changes in gene expression levels [51, 52]. rs1800734 has not clearly emerged from CRC GWAS, though a large study has provided evidence that this SNP is a risk factor for CRC in a study of 10,409 CRC cases and 6965 controls with a significant per allele odds ratio of 1.06 [23]. However, meta-analyses have not supported these findings [23, 24]. Regardless of its influence on overall CRC incidence, substantial evidence exists for association of rs1800734 with the MSI-H subtype of CRC [21, 22]. In addition to methylation changes, we have also previously shown functional changes incurred due to the variant SNP genotype. Specifically, promoter constructs with either the G or A allele were transfected into a variety of cell lines including the CRC and normal colonic cell lines [53]. The variant A allele exhibited significantly less luciferase activity than the G allele in all cell lines tested. We also established, through electrophoretic mobility shift assay experiments in the CRC cell line HCT 116 and normal colonic cell line CCD-841-CoTr, the presence of a DNA-binding factor(s) with high affinity for the G allele but not A [51]. This work was replicated in HeLa cell nuclear extract by others [54]. Active promoters bound by transcription factors and RNA polymerase II are more resistant to incurring DNA methylation than inactive promoters [55, 56]. Therefore, if transcription factors are unable to bind at the A allele, this likely provides the link between variant SNP genotype and increased CpG island methylation, but does not yet provide a mechanism for methylation changes at the shore.

These results have shown an association between a SNP and methylation at the *MLH1* CpG shore in normal colorectal tissue and PBMCs, in contrast to previous studies, which demonstrated a SNP-methylation association at the CpG island in tumour DNA. Tumour methylation of the CpG island is associated with MSI-H whereas shore methylation does not show any such association. The reasons for this are unclear. Perhaps the methylation changes at the CpG shore and CpG island are two independently regulated events. Validation of these findings in other large, well-characterized CRC cohorts would confirm these SNP-associated methylation events. Additional studies of the *MLH1* shore in other cancer and normal tissue types would also be of interest to determine whether this phenomenon is restricted to colorectal tissue.

We also observed stage-specific methylation changes at the *MLH1* shore in tumour DNA. Specifically, hypomethylation was seen among cases with stage IV CRC, despite the fact that across all cases tumour DNA incurs hypermethylation of the *MLH1* shore in comparison to PBMC and normal colorectal DNA. These differences were apparent in cases with each genotype of rs1800734. These results further demonstrate the dynamic methylation patterns of the *MLH1* shore with respect to tumour stage, as well as SNP genotype and tissue type. Methylation studies have generally focused on hypermethylation events in cancer; however, several have investigated hypomethylation in advanced CRC, such as at LINE-1 repeats [55, 56]. Future validation of these findings in a larger number of stage IV cases would be of value.

An advantage of this study is the availability of DNA from a large population-based cohort. This cohort is well established with DNA available from matched blood, normal colorectal tissue, and tumour, which enabled the ascertainment of a comprehensive view of methylation patterns. The methylation differences measured between genotypes were significant, but relatively subtle. For example, in normal colorectal tissue, methylation was 32.8% in GG individuals, 27.3% in GA, and 24.0% in AA. Despite this, the data still demonstrated a significant SNP association in just six samples used for the bisulfite sequencing experiments. Across multiple techniques in two types of normal DNA sources, whether a large or small sample size, the genotype-epigenotype association at the *MLH1* shore is significant.

A caveat to this study is that every CpG of the *MLH1* shore cannot be individually examined due to limitations of the techniques used. MethyLight is a real-time PCR-based method that is limited to approximately 150-base-pair amplicons. This technique is only able to detect methylation if all CpGs in the primer and probe sequences are methylated, and does not account for variable methylation patterns. Bisulfite sequencing will account for variable methylation patterns that MethyLight cannot, and one can sequence a region up to 300 base pairs. However, this method is low throughput and not well suited for analysis of a large number of samples. Despite differences in technique, we were still able to detect significant SNP-associated hypomethylation at the *MLH1* shore in normal DNA. Another limitation is that we only assessed methylation of the shore upstream of the *MLH1* CpG island. There is also a shore downstream of *MLH1* located within its coding region. We did not observe SNP-associated methylation changes in our previous methylation analysis of PBMCs in CRC cases or controls, thus we did not pursue this downstream shore in the current study [25].

## Conclusions

These results demonstrate an association between the promoter polymorphism rs1800734 and DNA hypomethylation at the *MLH1* shore in normal colorectal tissue, and also confirmed this in PBMCs, building upon our previous work. This association is not evident in tumour DNA from the same cases, but instead, as previously demonstrated, this polymorphism is associated with hypermethylation at the CpG island in MSI-H CRC. These results reveal that the epigenetic landscape of *MLH1* is dynamically regulated at least in part by the static genetic sequence. Additional characterization of epigenetic and/or transcriptional regulation at the *MLH1* CpG island and shore, taking into account rs1800734 genotype differences, may lead to further insight into mechanisms by which polymorphisms contribute to cancer risk.

## Abbreviations

CRC: Colorectal cancer
GWAS: Genome-wide association study
MLH1: MutL homolog 1
MMR: Mismatch repair
MSH2: MutS homolog 2
MSH6: MutS homolog 6
MSI: Microsatellite instability
MSI-H: Microsatellite instability high
MSI-L: Microsatellite instability low
MSS: Microsatellite stable
OFCCR: Ontario Familial Colorectal Cancer Registry
PBMC: Peripheral blood mononuclear cell
PCR: Polymerase chain reaction
PMR: Percent methylated reference
PMS2: PMS1 homolog 2, mismatch repair system component
SD: Standard deviation
SNP: Single nucleotide polymorphism
TCAG: The Centre for Applied Genomics
